# Novel Insights into Guide RNA 5′-Nucleoside/Tide Binding by Human Argonaute 2

**DOI:** 10.3390/ijms17010022

**Published:** 2015-12-24

**Authors:** Munishikha Kalia, Sarah Willkomm, Jens Christian Claussen, Tobias Restle, Alexandre M. J. J. Bonvin

**Affiliations:** 1 Institute of Molecular Medicine, University of Lübeck, Ratzeburger Allee 160, 23538 Lübeck, Germany; munishikakalia@gmail.com (M.K.); willkomm@imm.uni-luebeck.de (S.W.); 2 Institute for Neuro- and Bioinformatics, University of Lübeck, Ratzeburger Allee 160, 23538 Lübeck, Germany; j.claussen@jacobs-university.de; 3 Computational Structural Biology, Bijvoet Center for Biomolecular Research, Faculty of Science—Chemistry, Utrecht University, Padualaan 8, 3584 CH Utrecht, The Netherlands

**Keywords:** RNAi, MD, enzyme kinetics, pre-steady-state kinetics, fluorescence spectroscopy

## Abstract

The human Argonaute 2 (hAgo2) protein is a key player of RNA interference (RNAi). Upon complex formation with small non-coding RNAs, the protein initially interacts with the 5′-end of a given guide RNA through multiple interactions within the MID domain. This interaction has been reported to show a strong bias for U and A over C and G at the 5′-position. Performing molecular dynamics simulations of binary hAgo2/OH–guide–RNA complexes, we show that hAgo2 is a highly flexible protein capable of binding to guide strands with all four possible 5′-bases. Especially, in the case of C and G this is associated with rather large individual conformational rearrangements affecting the MID, PAZ and even the N-terminal domains to different degrees. Moreover, a 5′-G induces domain motions in the protein, which trigger a previously unreported interaction between the 5′-base and the L2 linker domain. Combining our *in silico* analyses with biochemical studies of recombinant hAgo2, we find that, contrary to previous observations, hAgo2 is capable of functionally accommodating guide strands regardless of the 5′-base.

## 1. Introduction

RNAi is a highly specific post-transcriptional mechanism, which regulates gene expression through gene silencing. RNAi was initially discovered in the nematode *Caenorhabditis elegans* [1]. RNAi plays crucial roles in developmental processes and maintenance of cellular as well as systemic homeostasis [2,3,4,5]. Thus far, more than 1800 human miRNAs have been identified [6]. It is assumed that 20%–30% of human genes are likely regulated by miRNAs [7,8,9,10]. Aberrant expression and/or function of miRNAs may cause several pathological states like various types of cancer as well as metabolic, neurodegenerative, infectious, and chronic inflammatory diseases [11,12,13].

RNAi is a multi-step reaction and RNA-induced silencing complex (RISC) formation is central to this process. RISC is a multi-protein complex that minimally consists of the Argonaute2 (Ago2) protein and a miRNA or siRNA guide strand. miRNAs are small RNAs that are ~22 nucleotides (nt) in length; they are known to ubiquitously regulate a wide range of eukaryotic cellular processes [14]. The double-stranded (ds) miRNA precursors are loaded into Ago2 with the thermodynamically less stable 5′-end strand preferred as a guide strand, while the passenger strand is released or degraded [15,16].

While the human Ago family consists of four members, hAgo1–4, all believed to be closely associated with small RNAs, only hAgo2 embodies an endonucleolytic “slicer” activity [17,18]. Thus far, a range of *Thermus thermophilus* Argonaute (TtAgo) structures, in different combinations of guide and target strands, have provided conclusive structural insights into the overall architecture of Argonaute proteins [19,20,21,22]. The full-length eukaryotic crystal structures of human and yeast *Kluyveromyces polysporus* Argonaute proteins revealed the eukaryotic counterparts [23,24,25,26,27,28]. Surprisingly, despite very low sequence identity (~12%) between hAgo2 and TtAgo, the architecture of the protein is highly conserved; this is one of the finest examples of domain conservation over sequence conservation [25].

hAgo2 is composed of four domains, N-terminal (N), PIWI-Argonaute-Zwille (PAZ), Middle (MID) and P-element induced wimpy testes (PIWI), tethered by two linker domains, L1 and L2 [23,25]. The structure is bilobed with a central cleft, which forms the nucleic acid binding channel. One lobe consists of the N and PAZ domains and the other is formed by the MID and PIWI domains. In hAgo2, eleven specific eukaryotic insertions were identified, compared to the prokaryotic counterparts [29]. Three of these insertions are located in the nucleic acid binding channel, which causes a lengthening of this structural feature. A specific eukaryotic C-terminal helical insertion was also revealed, which carries several phosphorylation sites and is believed to play some role in discriminating between a single stranded guide and a guide–target duplex [30].

The mode of small RNA binding to hAgo2 is comparable to the prokaryotic Agos: both ends of the guide RNA are fixed, the 5′-end by the MID domain and the 3’-end by the PAZ domain [19,20,21,23,25]. The guide strand sits in a nucleic acid binding groove, with protruding side chains of the neighboring protein residues introducing two major kinks in the RNA. In its course across the protein, the guide RNA interacts with all four hAgo2 domains together with the two linkers [23,25].

The 5’-end of the guide RNA forms a multitude of specific interactions with several residues in a tight binding pocket of the MID domain [25,31]. A distinct loop known as the nucleotide specificity (NS) loop, close to the MID binding pocket, binds the base of the 5′-nucleotide [31]. Sequence analysis performed on various nematode, fly, plant and conserved human miRNAs reveal that there is a strong bias for U or A at the 5′-position of the guide strand [31,32,33,34,35]. Crystal structures of the hAgo2 MID domain in the presence of nucleoside monophosphates mimicking the 5′-end of miRNAs shed some light on this bias. The hydrogen-bonding patterns of CMP and GMP are completely opposite to those of AMP and UMP. Furthermore, the amide group present in GMP collides with the carbonyl group of G524 present in the NS loop. NMR titration experiments of an isolated hAgo2 MID domain with nucleoside monophosphates further confirm this bias, as UMP and AMP have been shown to have 30-fold higher binding affinity in comparison to CMP and GMP [31].This bias supposedly plays a role in the loading of miRNA into Ago proteins with 5′-U strands being strongly preferred [36]. Further confirmation originates from studies showing that the target nucleotide opposite the guide 5′-nucleotide is bound within a binding pocket formed by the MIDand L2 domains of hAgo2 [28]. Biochemical analyses revealed an affinity up to three times higher for targets carrying an adenine in this position. This might enhance dwell times of binary hAgo2–guide complexes on seed-paired target RNAs [37].

In the present study, we combine molecular dynamics (MD) simulations of binary hAgo2/OH–guide–RNA complexes with biochemical studies using full-length recombinant hAgo2 and different model RNA substrates to analyze the reported discrimination among different guide 5′-nucleotides. We find hAgo2 to be highly flexible facilitating binding of guide strands comprising all four possible nucleotides at the 5′-end. Moreover, the different guides trigger subsequent target RNA cleavage with comparable efficiency. Interestingly, conformational changes triggered by the different guide strands not only affect the 5′-binding pocked but also the PAZ and N domain to different degrees.

## 2. Results

We performed MD simulations making use of a recently published crystal structure of hAgo2 in complex with a bound guide miRNA (PDB 4F3T), to investigate the reported bias of hAgo2 for U or A at the 5′-position of the guide RNA in mechanistic terms. The MD simulations performed are summarized in Table 1. To verify the *in silico* generated results, detailed steady- and pre-steady-state binding and cleavage experiments with recombinant hAgo2 were conducted.

**Table 1:** List of simulations performed. For details, see Materials and Methods.

Chemical System	Force Field	MD Production Run Length
hAgo2 apo–enzyme	Amber03 ILDN	100 ns
hAgo2_5’-U–guide	AMBER Parmbsc0	100 ns
hAgo2_5’-A–guide	AMBER Parmbsc0	100 ns
hAgo2_5’-C–guide	AMBER Parmbsc0	100 ns
hAgo2_5’-G–guide	AMBER Parmbsc0	100 ns

### 2.1. hAgo2 Is Stabilized by Protein–RNA Interactions

**Figure 1:**
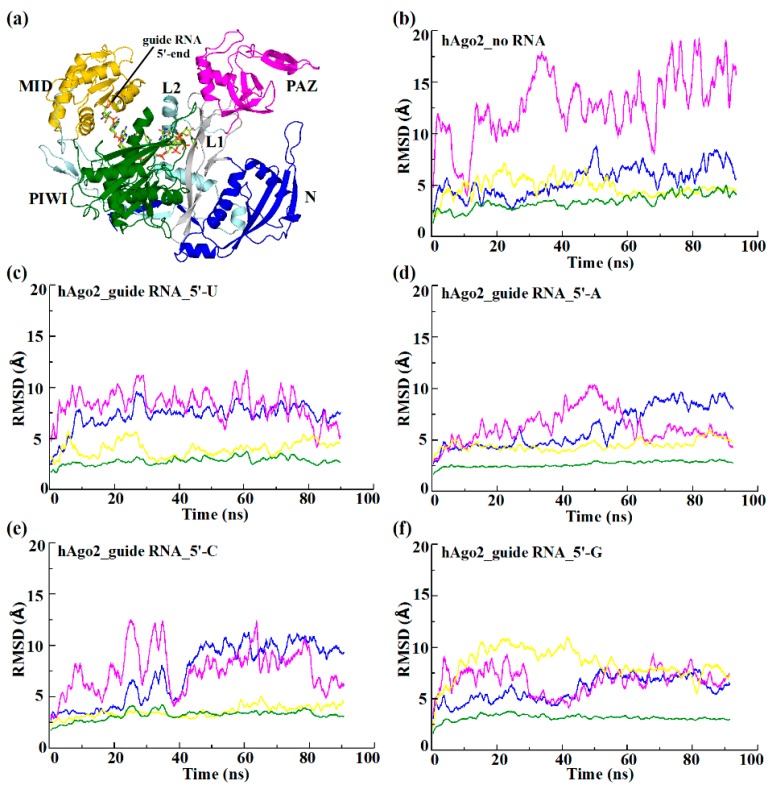
Analysis of backbone RMSD as a function of the simulation time for various hAgo2/guide RNA complexes. RMSD of the individual domains are calculated after superimposition on backbone atoms of the PIWI domain. (**a**) X-ray structure of full-length hAgo2 in complex with a truncated 10-mer guide miRNA (PDB 4F3T). The individual domains are labeled and color-coded: N (blue), PAZ (magenta), MID (gold) and PIWI (green) joined by two linker regions, L1 (silver) and L2 (pale cyan). The protein is represented in cartoon and the guide RNA in licorice; (**b**) Free hAgo2 in the absence of bound guide RNA; and hAgo2–guide RNA complexes with U (**c**); A (**d**); C (**e**); or G (**f**) at the 5′-end of the RNA.

MD simulations of the hAgo2–miR20a complex reveal a breathing motion of the protein caused by large movements of the N and PAZ domains (Figure 1). The movement and flexibility of the PAZ domain dominates this breathing motion, although the L1 and L2 linker regions seem to regulate and orchestrate it. The PAZ domain behaves like a pulley, tethered on both sides by the two-linker regions acting like a fictitious rope, which pulls the N domain on one side and the MID/PIWI domains on the other. Despite the large motions in the protein, the PIWI domain remains stable throughout, acting like the core of the protein. Moreover, MD simulations of hAgo2 in the absence of bound guide RNA confirm that the PAZ domain is the major contributor towards global protein flexibility. We observed that the root mean square deviation (RMSD) of the PAZ domain increased from ~5 Å to >15 Å in the absence of RNA, as shown in Figure 1b. This clearly indicates that the protein–RNA interactions are integral in stabilizing the hAgo2–guide RNA complex.

### 2.2. Different Guide 5′-Bases Induce Different Inter-Domain Motion Patterns in hAgo2

To study the effect of the four possible 5′-bases on the inter-domain motion of the hAgo2–guide RNA complex, MD simulations were performed where the 5′-U was replaced by A, C and G, respectively. The simulations revealed that individual 5′-base interactions differently affect the observed inter-domain motions of hAgo2.

In the presence of a 5′-U guide RNA, the hAgo2 domain motion is dominated by movements of the PAZ and N domains, while MID and PIWI remain relatively stable (Figure 1c). As we replace 5′-U with 5′-A, the overall inter-domain motion pattern of hAgo2 is comparable to the situation with 5’-U (Figure 1d). In the presence of a 5′-C, the inter-domain motion pattern changes and the protein becomes more flexible (Figure 1e). The motion and flexibility of the N domain noticeably becomes more explicit (Supplementary Material, Figure S1). However, hAgo2 retains its usual alternating breathing motion caused by large movements of the PAZ domain. The MID domain appears less flexible than observed for 5′-U or 5′-A, while the PIWI domain remains to be the least flexible of all the domains, retaining its function as the core of the protein. Surprisingly, in the presence of 5′-G, the inter-domain motion of hAgo2 is unique as compared to the other three bases showing an antagonistic domain motion pattern (Figure 1f). Here, especially extensive movements of the MID domain replace the conventional pattern dominated by movements of the PAZ domain. The RMSD of the MID domain increases up to ~8 Å (Figure 1f). Additionally, the domain undergoes a rather large conformational change (Supplementary Material, Figure S1). The PAZ and N domains also contribute to these domain movements, whereas the behavior of the PIWI domain remains unaltered. The RMSD means and standard deviations of individual domains of hAgo2 in presence of different 5’-bases, are summarized in Table 2.

**Table 2:** Mean and standard deviation of the RMSD calculated for hAgo2 protein domains in the presence of different 5′-bases (U, A, C, and G). The RMSD were calculated from the 100 ns MD simulations after fitting on backbone atom of each domain separately.

Simulation System Name	RMSD (Å)			
	N	PAZ	MID	PIWI
hAgo2_guide RNA_5′-U	0.72 ± 0.13	0.74 ± 0.18	0.42 ± 0.07	0.30 ± 0.03
hAgo2_guide RNA_5′-A	0.61 ± 0.20	0.62 ± 0.17	0.45 ± 0.06	0.26 ± 0.02
hAgo2_guide RNA_5′-C	0.73 ± 0.23	0.76 ± 0.23	0.35 ± 0.06	0.32 ± 0.05
hAgo2_guide RNA_5′-G	0.59 ± 0.12	0.67 ± 0.14	0.84 ± 0.16	0.32 ± 0.03

**Figure 2:**
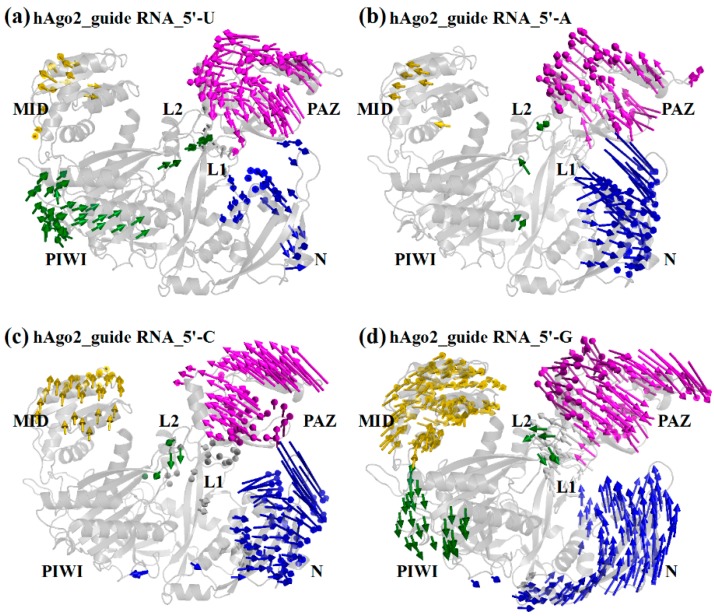
PCA analysis on backbone atoms of hAgo2/guide RNA complexes with different 5′-bases. For clarity, the nucleic acid is not shown. (**a**) U; (**b**) A; (**c**) C; and (**d**) G at the 5′-position of the guide RNA. Color-coding is given in Figure 1.

The specific inter-domain protein motion pattern of hAgo2 is corroborated by the Principal Component Analysis (PCA) of the trajectories in the presence of the respective 5′-bases. PCA was performed for the entire protein backbone. For each of the different 5’-bases a distinct pattern is observable. In the presence of a 5′-U the most pronounced domain motion is observed for the PAZ domain (Figure 2a). The domain motion of hAgo2 is similar in the presence of 5′-A with a correlated motion of PAZ and parts of the N domain (Figure 2b). In the presence of 5′-C the motion of the N domain becomes more prominent than that of the PAZ domain (Figure 2c). Yet, the most striking motion occurs in the presence of 5′-G, where the MID domain shows an unusually high domain motion, not observed for any of the other 5′-bases (Figure 2d).

**Figure 3:**
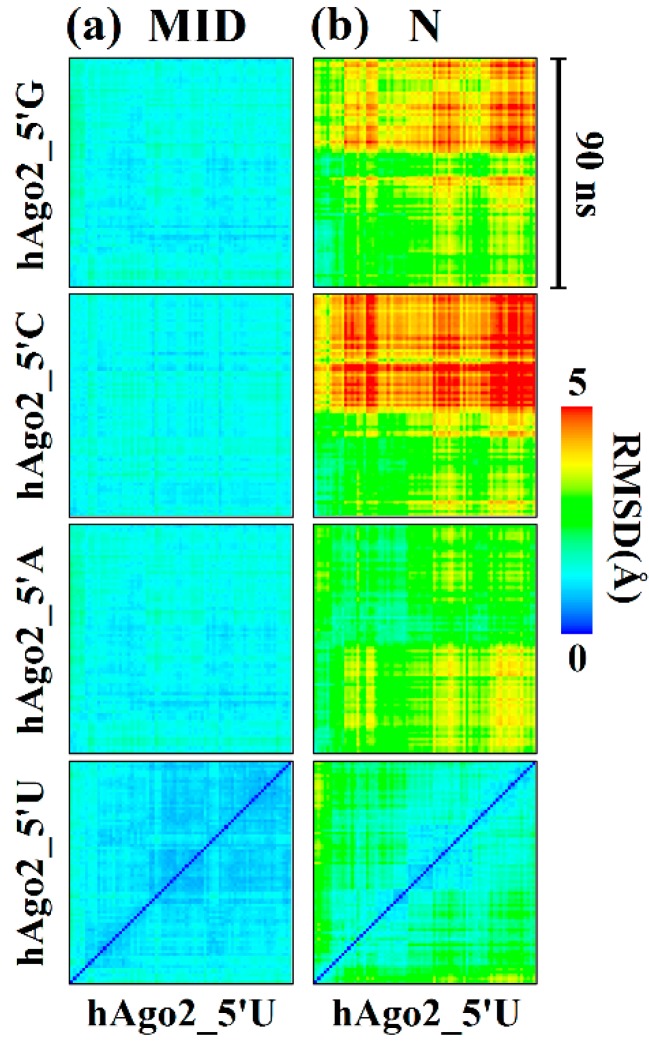
Cross-RMSD plots of hAgo2 MID (**a**); and N-terminal (**b**) domain. Cross-RMSD were calculated after superimposition on backbone atoms of the MID and N domains. The different 5′-bases are indicated on the left. While the RMSDs (color-coded with a gradient from 0 to 5 Å) reveal very little changes in the MID domain between the various 5′-end guides, a clear difference can be observed in the N-terminal domain with gradually increasing RMSDs when switching form 5′-U to 5′-G end guides.

Differences in inter-domain motions of hAgo2 between the various systems were visualized with the aid of cross-RMSD plots by comparing the 5′-U simulation with all other systems. The cross-RMSD plot for the MID domain (Figure 3a) shows that this domain is stable and does not show much domain motion. However, a clear difference was observed for the N domain (Figure 3b). The cross-RMSD values for the N domain constantly increase as we replace 5′-U with 5′-A, 5′-G and 5′-C. This implies that the 5′-base directly influences the motion of the N domain.

### 2.3. Steady-State and Pre-Steady-State Binding Experiments of hAgo2 with Different Guide and Target Substrates

**Figure 4:**
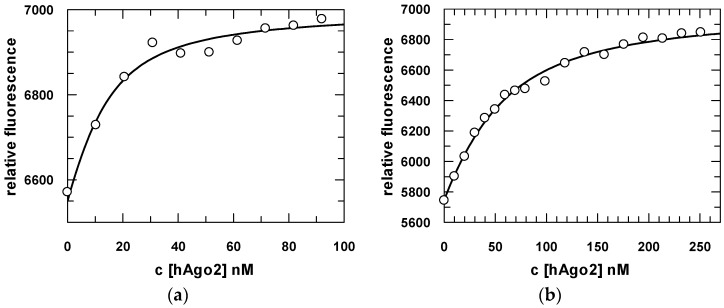
Fluorescence equilibrium titrations of hAgo2 with guide RNA. 20 nM of FAM-labeled guide RNA carrying a 5′-G (s2b-FAM) without (**a**) or with (**b**) a phosphate group were titrated with increasing amounts of hAgo2. Data were fitted to a quadratic equation and a representative fit is shown. *K_d_*s of 7.5 ± 1.8 nM without a phosphate group and 34.7 ± 2.8 nM with a phosphate group at the guide 5′-end were obtained.

We characterized the binding affinity as well as association and dissociation kinetics of various binary (hAgo2/guide RNA) or ternary (hAgo2/guide/target RNA) complexes with steady-state and pre-steady-state binding experiments. For a 21-mer 5′-U RNA guide, we had previously determined *K*_d_ values for binary complexes of 7 nM in the case of a 5′-phosphorylated and 106 nM in the case of an un-phosphorylated substrate [38]. Interestingly, here we observe quite the opposite for a 5’-G guide: the 5′-phosphorylated substrate shows a binding affinity of about 35 nM, whereas the un-phosphorylated RNA binds with about 7 nM (Figure 4). The 5′-A and 5′-C guides show affinities of in each case about 5 nM for the 5′-phosphorylated and 8 nM and 7 nM for the un-phosphorylated version, respectively. (Supplementary Material, Figure S2).

Performing transient binding experiments using a stopped flow device, we are able to dissect the protein/RNA interaction in particular sub-steps [38]. Assembly of a binary hAgo2/guide RNA complex comprises at least three kinetically distinct binding steps, with the first step (*k*_+1_) representing a diffusion-limited collision complex formation. The second and third steps (*k*_+2_ and *k*_+3_) represent anchoring of the guide strand 5′-end into the Mid binding pocket and binding of the guide strand 3′-end to the PAZ domain, respectively. The corresponding dissociation rate constants (*k*_−1_, *k*_−2_ and *k*_−3_) reflect the back-reaction. Accordingly, one would expect that especially motions of the MID or PAZ domain might be reflected by changes of the association or dissociation rate constants of the second and/or third step of binary complex formation; *i.e.*, *k*_+2_/*k*_−2_ or *k*_+3_/*k*_−3_. Interestingly, we indeed do observe slight changes upon 5'-U *versus* 5'-G guide/hAgo2 binary complex dissociation while complex association is not affected (Supplementary Material, Figure S3). While *k*_−2_ is accelerated by a factor of about 3, *k*_−3_ at the same time is reduced by a factor of about 3. The corresponding data are summarized in Table 3. This would imply that domain motions triggered by different 5′-bases as shown in Figure 2 do, albeit to a small extent, affect transient hAgo2/guide RNA interactions. In the case of ternary complex formation (hAgo2/guide/target), no differences between different 5’-bases could be observed (data not shown).

**Table 3:** Summary of equilibrium and pre-steady-state binding data for hAgo2/G–guide RNA complex formation. Numbers in the first row represent equilibrium measurements (*K*_d_meas_). Numbers in the second column were calculated from the corresponding association and dissociation rate constants (*K*_d_cal_ = *k*_off_/*k*_on_ = (*k*_−1_/*k*_1_) × (*k*_−2_/*k*_2_) × (*k*_−3_/*k*_3_)). Differences in *K*_d_ values for binary complexes determined via equilibrium and pre-steady-state measurements most probably arise from a slight protein aggregation causing a reduced diffusion rate by 2–3-fold leading to an underestimating of *k*_+1_bin_ and thus higher *K*_d_ values. The numbers given are an average of at least two independent experiments. Most experiments were repeated up to three or four times. Standard deviations are given in brackets. * Data taken from [38]. Details are given in the text.

Substrate	*K*_d_meas_ (nM)	*K*_d_cal_ (nM)	*k*_1_ (M^−1^·s^−1^)	*k*_−1_ (s^−1^)	*k*_2_ (s^−1^)	*k*_−2_ (s^−1^)	*k*_3_ (s^−1^)	*k*_−3_ (s^−1^)
U–guide *	7 ± 0.9	36	0.6 (±0.001) × 10^8^	6.2 ± 0.6	0.26 ± 0.02	0.17 ± 0.02	0.012 ± 5 × 10^−4^	0.007 ± 1 × 10^−4^
G–guide	35 ± 0.4	59	0.16 (±0.006) × 10^8^	2 ± 0.6	0.2 ± 0.06	0.54 ± 0.07	0.02 ± 0.01	0.0023 ± 4 × 10^−4^
OH-G–guide	7 ± 0.5	21	0.27 (±0.005) × 10^8^	2.1 ± 0.08	0.3 ± 0.2	0.6 ± 0.02	0.013 ± 0.002	0.002 ± 7 × 10^−4^

### 2.4. Cleavage Assay with a 5’-G Guide RNA

**Figure 5:**
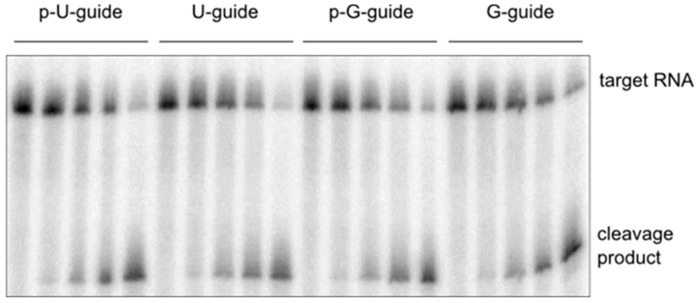
hAgo2-mediated cleavage of target RNA with guide strands carrying either a 5’-uridine or a 5’-guanosine with or without a 5′-phosphate. Cleavage assays were conducted using 2.5 µM hAgo2, 100 nM guide RNA (as2b or G-as2b) and 2.5 nM target RNA (ICAM-1-IVT). Samples were taken at time points 0ʹ, 5ʹ, 25ʹ, 55ʹ, and 120ʹ and analyzed using 8% denaturing PAGE. Detection was carried out via autoradiography.

Besides binding experiments, we also performed cleavage assays with a 21-mer guide RNA bearing either 5′-phosphorylated or un-phosphorylated 5′-G in combination with a short 21-mer target RNA fully complementary to the guide. As shown in Figure 5 the observed cleavage reactions look the same. Moreover, the amplitude and the cleavage pattern were virtually indistinguishable from results obtained previously with a 5′-U guide [38]. A similar cleavage pattern could also be observed for 5′-A and 5′-C guides (data not shown). This finding evidently suggests while the guide 5'-binding pattern might be different the overall Ago2/guide interaction is the same otherwise one would not expect to observe an identical cleavage pattern.

## 3. Discussion

While previous X-ray studies [31] have focused on the interaction of nucleoside monophosphates with an isolated hAgo2 MID domain, here we were interested in the interaction of full length protein and its corresponding guide strand. The starting point for our studies was a recently published co-crystal structure of hAgo2 with a miRNA–20a guide RNA [25]. In addition to MD simulations of different binary hAgo2/guide RNA complexes, we performed detailed biochemical studies to complement our *in silico* data.

The co-crystal structures of the hAgo2 MID domain with different nucleoside monophosphates (PDBs 3LUK, 3LUD, 3LUG and 3LUH) reveal that 5′-UMP and 5′-AMP have different interaction patterns from that of 5′-CMP and 5′-GMP. The bases of 5′-UMP and 5′-AMP form specific hydrogen bonds with the backbone of T526 and G524, while 5′-CMP and 5′-GMP do not form hydrogen bonds, since the amide group present in the pyrimidine ring of 5′-GMP would clash with the carbonyl group of G524. This interaction pattern has been used to explain the bias of hAgo2 towards 5′-U and 5′-A and suggests that the human Ago family might go to great lengths to discriminate different guide 5’-bases. It is tempting to speculate that hAgo proteins evolved in a manner to preferentially accommodate miRNAs with 5′-U over other 5′-bases based on the sheer abundance of the human miRNAs with 5′-U [34].

**Figure 6:**
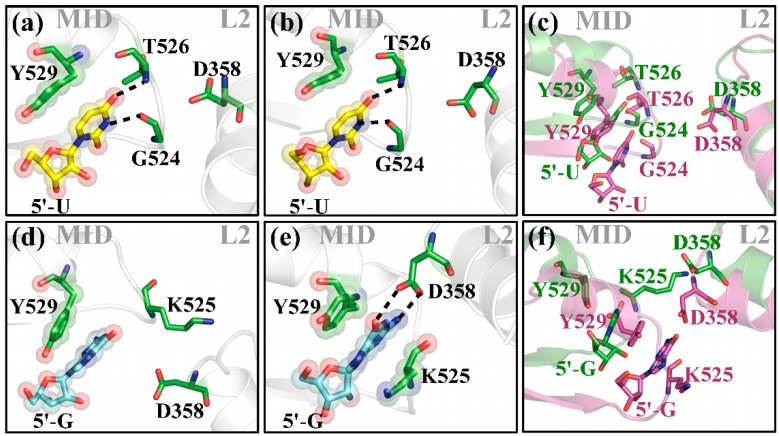
Close-up of the guide RNA 5'-end and relevant protein residues of the MID domain and L2 linker region. The MID domain and the L2 helix are represented in cartoon (grey); important protein residues (green); and 5′-U (yellow); or 5′-G (blue) are shown in sticks. For clarity, merely the terminal base is shown. Hydrogen bonds are represented by black dotted lines. Transparent spheres highlight stacking interactions between protein residues and the corresponding bases. (**a**,**d**) show the initial situation; and (**b**,**e**) after 20 ns of simulation of 5′-U and 5′-G terminated hAgo2 bound guide strands, respectively; (**c**,**f**) superposition of (**a**,**b**); and (**d**,**e**) with neighboring protein residues at *t* = 0 ns are shown in green and at *t* = 20 ns in magenta.

In agreement with the X-ray studies mentioned above, we observed from our MD simulations with full length protein bound to a 10-mer guide RNA that 5′-U and 5′-A ends retain the initial interaction pattern with the NS loop through the entire length of the simulation (Figure 6a,b and Supplementary Material, Figure S4a,b). In the case of a 5′-C this interaction pattern is stabilized by the formation of a novel hydrogen bond formation with the Q548 side chain (Supplementary Material, Figure S4d,e). However, the most striking interaction pattern was observed between 5′-G and the neighboring protein residues. We noticed that the MID domain undergoes a large domain movement at ~20 ns of the simulation, tilting the MID domain and bringing the NS loop in close proximity of the L2 region. This movement triggers hydrogen bond formation between the NH2 and carbonyl group of the 5’-G base and OD1 and OD2 of ASP358 present in helix7 of the L2 region. Once the hydrogen bond formation occurs, it is retained thereafter for the entire remaining of the simulation. In addition to this hydrogen bond interaction, perfect alignment of the aromatic rings of the base with K525 side chain occurs, leading to a cation–π interaction. This cation–π interaction complements the base stacking that already exists between the aromatic ring of the 5′-G and the Y529 side chain (Figure 6e). Y529 represents a highly conserved residue that has been shown to play a critical role in guide RNA binding and, if phosphorylated, drastically reduces such interactions [39]. This novel cation–π interaction in addition to the stacking interaction between Y529 and 5′-G pyrimidine ring could explain the observed unexpected high binding affinity of about 7 nM of hAgo2 towards an un-phosphorylated 5′-G guide RNA (Table 3). Binding affinity for the phosphorylated guide is about 34 nM. Interestingly, quite the opposite is true for a 5’-U guide. Here the phosphorylated strand shows a binding affinity of about 7 nM whereas the un-phosphorylated version binds about 15-fold weaker. Obviously, in the case of 5'-U guides, hAgo2 discriminates strongly against the un-phosphorylated RNA. As to why this has evolved in such a way we currently can only speculate. It might have to do with the sheer abundance of human miRNAs with 5′-U. For the remaining two bases A and C no differences in binding affinity depending on the phosphorylation status could be observed. As reflected by the binding experiments, the different guide strands, regardless if 5′-phosphorylated or not, do not affect the hAgo2-mediated target RNA cleavage activity nor the observed cleavage position.

Interestingly, there appears to be an inbuilt mechanism in hAgo2, which enables binding of all four different guide 5′-bases; it is not restricted to the MID binding pocket, but the entire protein, with the exception of the PIWI domain, aids in this binding process by base-specific antagonistic domain motions. Those inter-domain motions are especially pronounced in the presence of 5′-C and 5′-G guide strands. The unique behavior of hAgo2 in the presence of 5′-G is very intriguing since it is completely opposite to that of 5′-U. The PAZ domain is by far the most flexible domain of all and contributes the most to different guide RNA binding patterns. The flexibility of the PAZ domain has been documented previously [40,41], while conformational changes within the N and MID domains have rarely been discussed.

Another interesting finding of this study is the novel interaction pattern observed between the 5′-G of the guide RNA and the L2 linker which occurred after ~20 ns into the simulation. It is interesting to note that for the co-crystal structure of the hAgo2 MID domain with 5′-GMP, residual electron density was observed only for the phosphate and ribose of the nucleotide, whereas the electron density of the base was notably missing [31]. This implies that a lack of stabilizing interactions between 5′-G and the NS loop results in dynamic disorder in the crystal. The MID domain experiences a domain movement, which brings it in the close proximity of helix7 of the L2 region instigating a hydrogen bond formation between the 5′-G and the D358 side chain. This suggests that hAgo2 has to undergo additional domain movements and conformational changes in order to accommodate the 5’-G in the most stable orientation. Once this arrangement is achieved, the 5′-G of the guide strand remains in this stable orientation. One of the prerequisites for this interaction is the inward movement of the MID domain towards helix7 of the L2 region. Since the 5′-end of the RNA is tightly bound to the MID binding pocket the guide is also pushed deeper into the nucleic acid binding channel, which in effect could influence the positioning and orientation of the incoming target strand. On the other hand, our biochemical cleavage experiments do not support such a scenario. Then again, the determined cleavage rate reflects the rate-limiting step of product release and thus might not be suited to monitor conformational rearrangements affecting the chemical step [38].

In summary, combining MD simulations of binary hAgo2/OH–guide–RNA complexes and biochemical studies with recombinant hAgo2, we observe considerable structural protein rearrangements in order to accommodate the corresponding 5′-bases. While thermodynamics of the protein/nucleic acid substrate interactions is not affected, small changes in the dissociation kinetics of the complexes are noticeable which nicely match in particular the identified structural transitions of the MID and PAZ domains.

## 4. Experimental Section

### 4.1. MD System Preparation

The crystal structure of hAgo2 (pdb: 4F3T) was used as starting point for all simulations. The missing loop residues, excluding the unstructured N-terminus, were modelled using Modeller9v10 [42]. The structure was first energy minimized in vacuum over 50,000 steps, applying the steepest descent method. It was then submerged in a box of 50,499 TIP3P water molecules with periodic boundary conditions. Twenty-three counter ions were added to neutralize the system, leading to a total of 150,703 atoms in each system. The solvated system was minimized over 50,000 steps and then equilibrated in two 100 ps simulations under NVT and NPT conditions, respectively, with position restraints on the solute. For temperature coupling during the NVT equilibration, the v-rescale thermostat was used. During NPT equilibration, the v-rescale thermostat and Berendsen barostat were used for regulating the temperature and pressure of the system. The equilibration of the system was performed at a reference temperature of 300 K. The position restraints on the solute were then released to start the production run. Non-bonded interactions were calculated using the Particle-Mesh-Ewald (PME) method with an order of 4 and a Fourier spacing of 0.16. The non-bonded cutoff for the van der Waal (vdW) interactions was set to 1.0 nm. Production runs of ~100 ns were performed for each system with a 2 fs integration time step. All MD simulations were performed with the GROMACS 4.6 simulation package using either the Amber03 ILDN (apoenzyme) or the AMBER Parmbsc0 force field (nucleic acid bound enzyme) [43,44]. The first simulation was carried out for the hAgo2 protein alone in the absence of guide RNA. The second simulation was carried out for the hAgo2 in complex with a truncated guide RNA (nucleotides 1–10, 5′-UAAAGUGCUU). In subsequent simulations, the U at the 5’-end of the guide RNA was replaced by A, C or G, respectively.

### 4.2. Protein Expression and Purification

Recombinant full-length hAgo2 was expressed and purified as described recently [38].

### 4.3. Oligonucleotides

All oligonucleotides were obtained from IBA (Göttingen, Germany) or Biomers (Ulm, Germany). Sequences of RNAs used for equilibrium fluorescence titrations, pre-steady-state stopped flow measurements and cleavage assays are listed in Table S1 (Supplementary Material). Where appropriate, RNAs were 5′-phosphorylated, either with [γ-^32^P] ATP (Hartmann, Braunschweig, Germany) or unlabeled ATP (Thermo Fisher Scientific, Waltham, MA, USA). In all cases, modified RNAs were purified by using Sephadex-G-50 columns (GE Healthcare, München, Germany), phenol/chloroform extraction, and ethanol precipitation.

### 4.4. Equilibrium Fluorescence Titrations

The binding affinity of recombinant hAgo2 in binding buffer (10 mM Tris, pH 7.5, 100 mM KCl, and 0.5 mM MgCl_2_) to the different guide RNA strands was determined in a 700-μL cuvette, where either FAM-labeled substrates (5′-U or 5′-G guide RNA; 20 nM each) or a binary complex (600 nM hAgo2/20 nM 5′-phosphorylated 5′-U guide RNA (as2bFAM)) were titrated with increasing concentrations of hAgo2 or a competitor 5’-A or 5’-C guide RNA, and change in fluorescence signal was recorded by using the FluoroMax-3 fluorescence spectrometer (Horiba Jobin Yvon, Bensheim, Germany). The samples were excited at 490 nm, and the emission intensity was measured at 520 nm. Slits were set to 1 nm. The experimental data were either fitted to a quadratic equation using the program GraFit 5, $Fluorescence=F_{max}-∆F_{max}\times \frac{\frac{\left(S+E+K_{d}\right)}{2}-\sqrt{\frac{{\left(S+E+K_{d}\right)}^{2}}{4}-S\times E}}{S}$, where *F*_max_ is the maximum fluorescence, ∆*F*_max_ is the maximal fluorescence change, S is the concentration of substrate, *E* is the concentration of the enzyme, and *K*_d_ is the equilibrium dissociation constant, or in the case of competitive titrations, they were evaluated by using the program Scientist (MicroMath Scientific Software, Saint Louis, MO, USA), which allows the user to define the system under investigation as a series of parallel equations defining (in this case) each discrete equilibrium, the relationship between the total and free concentrations of the components, and the way in which the observable signal is generated.

### 4.5. Pre-Steady-State Stopped-Flow Measurements

For binary complex formation (hAgo2 and siRNA substrates), 20 nM fluorophore-labeled guide RNA was rapidly mixed in binding buffer with hAgo2 (400–700 nM) and the change in fluorescence signal was recorded over time using the stopped flow instrument SX20 (Applied Photophysics, Leatherhead, UK). For the dissociation of binary complexes, 20 nM fluorophore-labeled guide RNA was preincubated with 600 nM hAgo2 and then rapidly mixed with a 100-fold excess of non-labeled competitor RNA. Excitation of the FAM-labeled RNAs was at 492 nm with slits set to 1 mm (equivalent to a wavelength bandwidth of 4.65 nm) and detection was through a filter with a cut-off at 530 nm. Reaction rates *k*_+*n*_ and *k*_−*n*_ were calculated by fitting the experimental data to an exponential equation using the program GraFit 5: Fluorescence = $\sum^{}A_{n}\;\times \;e^{\left(-k_{n}\;\times \;t\right)}$, where *A*_n_ is the amplitude corresponding to the observed phase, *k*_n_ is the rate constant of the observed phase and *t* is the time.

### 4.6. RNA Cleavage Assay

hAgo2 (2.5 μM) was incubated with 100 nM guide RNA (as2b or G-as2b) and 2.5 nM radiolabeled target RNA (ICAM-1-IVT) at 37 °C for up to 2 h in 10 mM Tris, pH 7.5, 100 mM KCl, 2 mM MgCl_2_, and 1 μg/mL RiboLock RNase Inhibitor (Fermentas). Reactions were stopped by addition of 1 vol 95% formamide, 0.025% (*w*/*v*) SDS, 0.025% (*w*/*v*) bromophenol blue, 0.025% (*w*/*v*) xylene cyanol, 0.5 mM EDTA, followed by PAGE analysis and autoradiography.

## 5. Conclusions

Our studies revealed that hAgo2 is a very flexible enzyme capable of functionally binding to guide strands carrying each of the four possible nucleobases at the 5’-end. This result is surprising and contradicts to some extent earlier observations made by others. However, one should consider the present study was performed with full length protein and a 10-mer (MD simulations) or 21-mer (biochemical studies) guide strand, while in other studies, an isolated MID domain and nucleoside monophosphates were used. As such, these studies are not really comparable. While the simulations with 5′-U and 5′-A guides closely resemble previous X-ray structures, hAgo2 has to undergo extensive rearrangements in order to accommodate 5′-C and 5′-G guides as one would propose from the aforementioned X-ray structures. Even though the MD simulations were performed in the absence of a 5′-phosphate group, our biochemical studies (e.g., binding studies of G–guide *versus* OH-G–guide) are in strong support of the *in silico* analyses. Interestingly, only in the case of a 5′-U guide the 5′-phosphate leads to a strong bias. Whether or not this has anything to do with the abundance of the human miRNAs with 5′-U, we can currently only speculate. Of the four protein domains, PAZ is the most flexible. Despite this, our MD simulations indicate that varying the guide 5′-nucleobase affects the entire protein rather than just the MID domain where 5′-binding occurs. Then again, according to our biochemical studies, such conformational rearrangements do not seem to interfere with enzyme function. This observation might not be too surprising considering the additional extensive conformational rearrangements upon formation of a catalytically active ternary complex as can be derived from biochemical studies of hAgo2 [38,45] as well as bacterial Agos [46,47], and be seen in different TtAgo/nucleic acid co-crystal structures [20,21].

## Supplementary Materials

Supplementary materials can be found at http://www.mdpi.com/1422-0067/17/1/22/s1.
