# Supplementary material for: Novel Insights into Guide RNA 5′-Nucleoside/Tide Binding by Human Argonaute 2
Source: Int J Mol Sci. 2015 Dec 24;17(1):22. doi: 10.3390/ijms17010022 (PMC4730269; doi:10.3390/ijms17010022)
Supplement: Supplementary file 1 [file ijms-17-00022-s001.pdf]

Supplementary Materials: Novel Insights into Guide RNA 5′-Nucleoside/Tide Binding by Human Argonaute 2

Munishikha Kalia, Sarah Willkomm, Jens Christian Claussen, Tobias Restle and Alexandre M. J. J. Bonvin

Table S1. Sequences of RNA oligonucleotides and *in vitro* transcript used in the present study. Bold face letters represent position of the C6-linked 5/6-FAM-label. The as2b-matching sequence in ICAM-1-IVT is underlined. Unless indicated otherwise, all strands were 5'-phosphorylated. * position of FAM-label

RNA	Sequence
as2b	5′-UAGAGGUACGUGCUGAGGCdTdT-3′
as2b-FAM	5′-UAGAGGUACGUGC**T***GAGGCdTdT-3′
A-as2b	5′-AAGAGGUACGUGCUGAGGGdTdT-3′
G-as2b	5′-GAGAGGUACGUGCUGAGGGdTdT-3′
s2b	5′-GCCUCAGCACGUACCUCUAdTdT-3′
s2b-FAM	5′-GCCUCAGCACGUACCUCUAdTd**T***-3′
slam	5′-CUGGACUUCCAGAAGAACAdTdT-3′
ICAM-1-IVT	5′-GGGCGAAUUGGGCCCGACGUCGCAUGCUCCCGGCCGCCAUGGCCG CGGGAUUAGCCGCAGUCAUAAUGGGCACUGCAGGCCUCAGCACGUACC UCUAUAACCGCCAGCGGAAGAUCAAGAAAUAAAUCACUAGUGCGGCC-3'

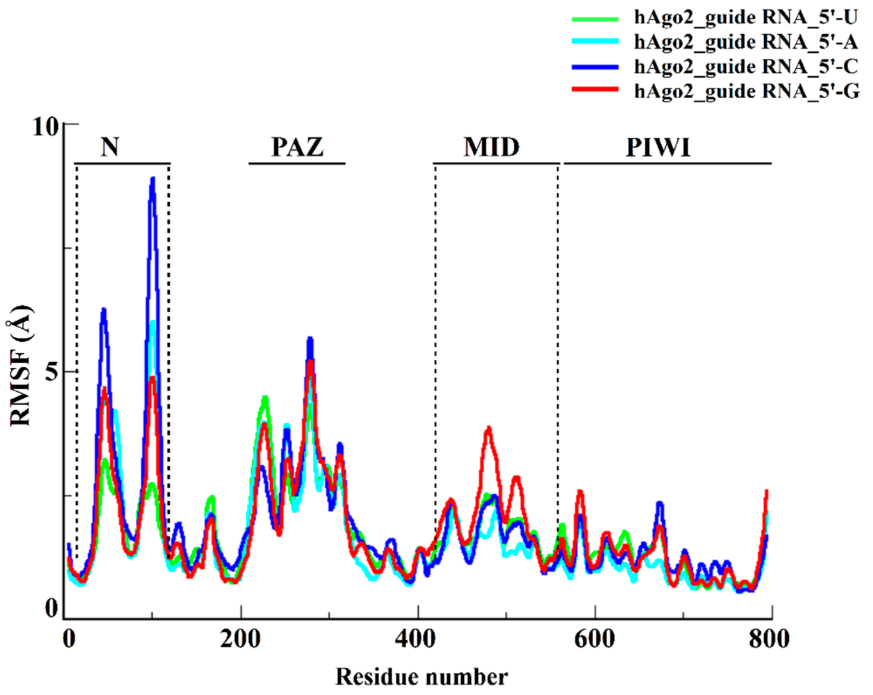

Figure S1. Root mean square fluctuations (RMSF) analysis of the hAgo2 in presence of different 5′-bases. The RMSF is calculated for the C-α atoms of the hAgo2 in the presence of 5′-U (green); 5′-A (cyan); 5′-C (blue); and 5′-G (red). The flexibility of the N and MID domains is highlighted by black dotted lines.

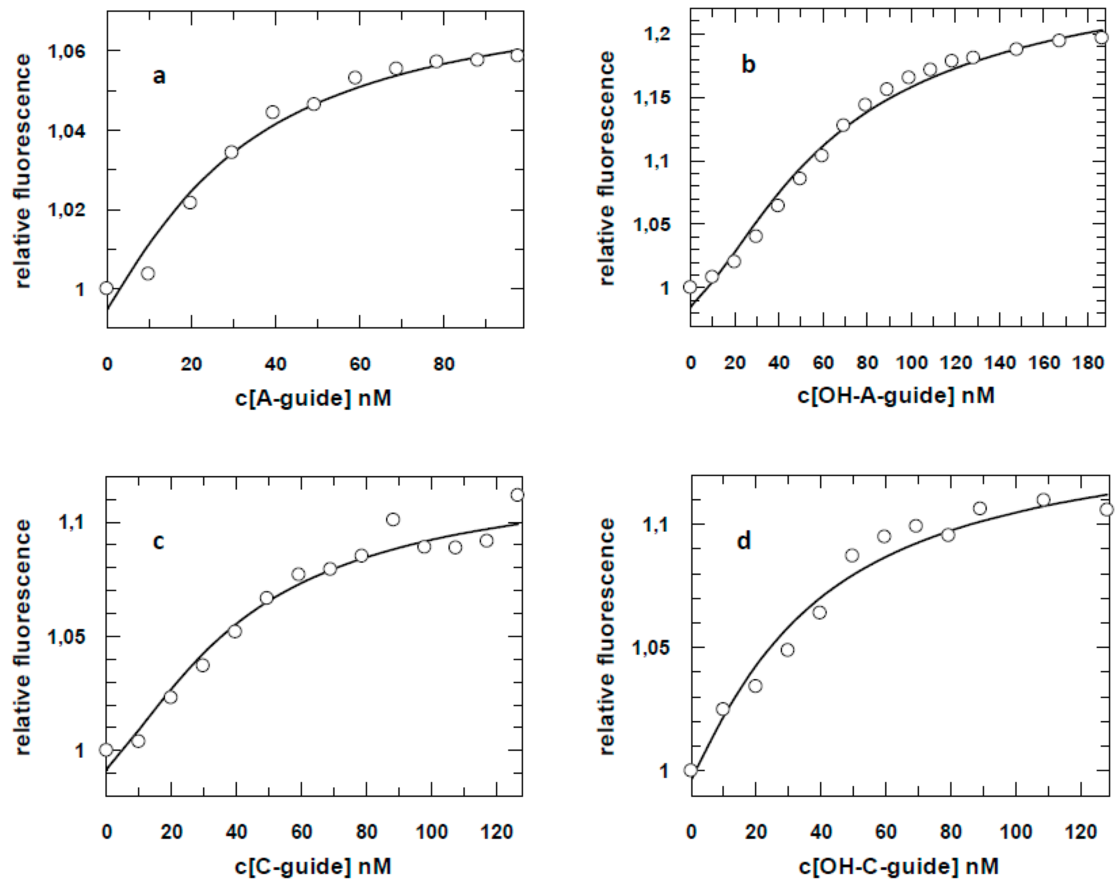

Figure S2. Displacement titration of fluorescent binary hAgo2-guide complexes with unlabeled guide RNAs. Binary complexes composed of 600 nM hAgo2 and 20 nM FAM-labeled guide RNA (as2b-FAM) were titrated with increasing concentrations of guide RNA carrying either an adenosine (A-as2b) in presence (**a**); or absence (**b**) of a phosphate group at the 5′-end or a cytidine (slam) in presence (**c**); or absence (**d**) of phosphate group at the 5′-end. The curves show the best fit by least-squares fitting to a model describing the two binding equilibria from which dissociation constants for the different guide strands were calculated: (**a**) 5.0 ± 1.9 nM; (**b**) 8.2 ± 2.0 nM; (**c**) 4.9 ± 2.2 nM (**d**) 6.6 ± 2.3 nM.

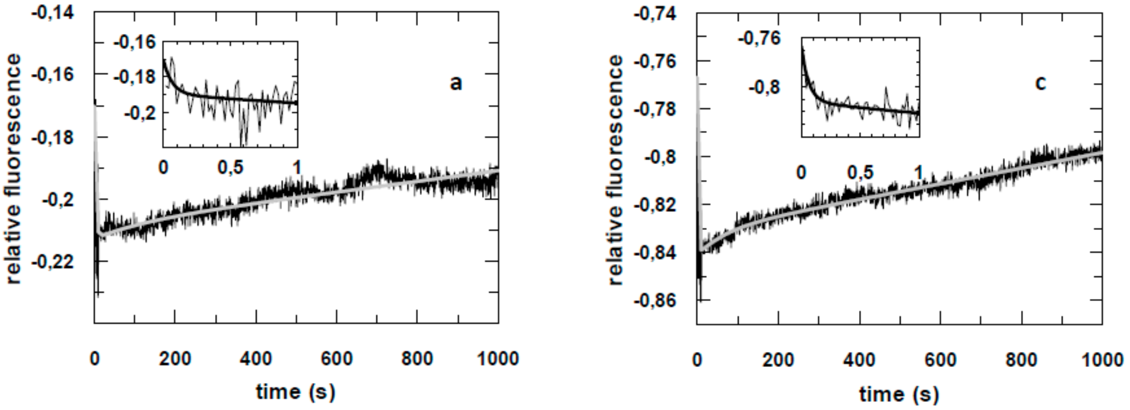

Figure S3. *Cont.*

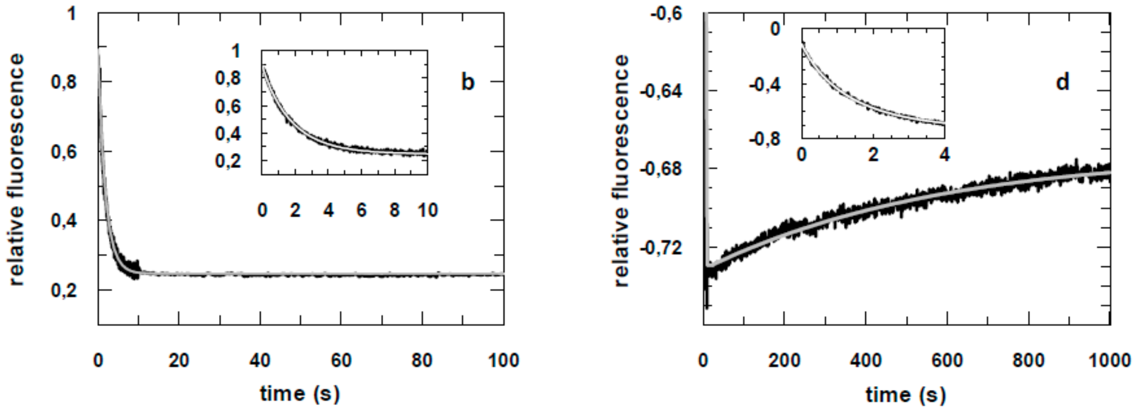

Figure S3. Pre-steady state kinetics of binary complex association and dissociation. Representative graphs are shown and the inserts show the reaction on a shorter time scale. Binding of 600 nM hAgo2 to 20 nM FAM-labeled guide (s2b-FAM) with a 5′-guanosine in presence (**a**); or absence (**b**) of a phosphate at the 5'-end of the guide. To monitor dissociation of binary complexes 600 nM hAgo2 were preassembled with 20 nM FAM-labeled guide (s2b-FAM) with a 5′-guanosine in presence (**c**); or absence (**d**) of a phosphate at the 5′-end of the guide. These binary complexes were competed with an excess of unlabeled guide as a competitor (as2b). In all four cases data could be fitted best to an equation with three exponential terms yielding the following rate constants: (**a**) k_1: 15.3 ± 2.7 s^{-1}; k_2: 0.26 ± 0.005 s^{-1}; k_3: 0.008 ± 0.001 s^{-1} (**b**) k_1: 16.5 ± 2.1 s^{-1}; k_2: 0.41 ± 0.03 s^{-1}; k_3: 0.014 ± 0.002 s^{-1} (**c**) k_1: 2.0 ± 0.2 s^{-1}; k_2: 0.54 ± 0.002 s^{-1}; k_3: 0.003 ± 0.0008 s^{-1} (**d**) k_1: 2.1 ± 0.08 s^{-1}; k_2: 0.61 ± 0.002 s^{-1}; k_3: 0.002 ± 0.00007 s^{-1}. The data are summarized in Table 2.

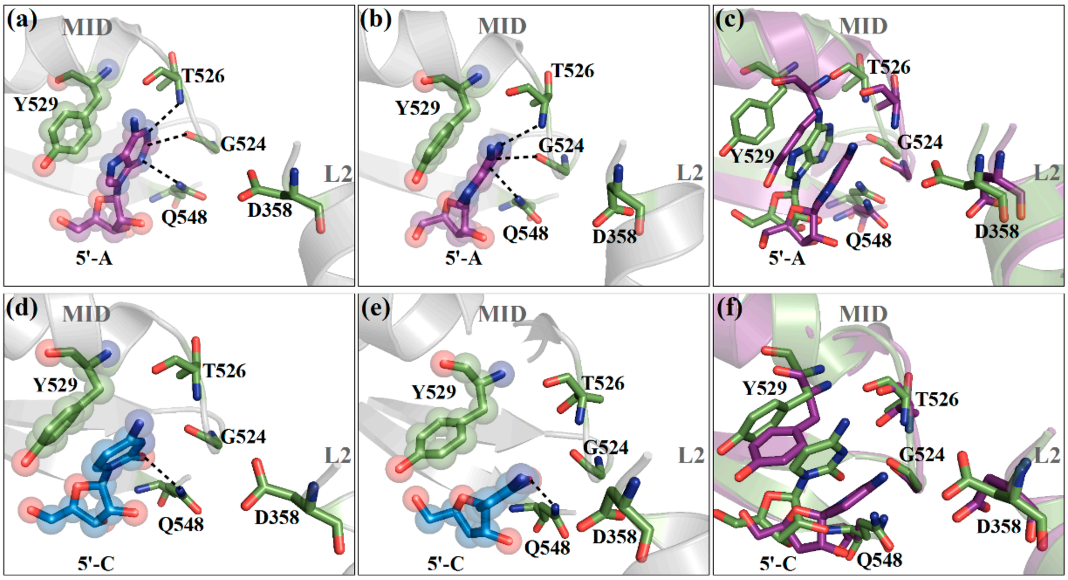

Figure S4. Close-up of the guide RNA 5′-end and relevant protein residues of the MID domain and L2 linker region. The MID domain and the L2 helix are represented in cartoon (grey), important protein residues (green) and 5′-A (purple) or 5′-C (dark blue) in sticks. For clarity merely the terminal base is shown. Hydrogen bonds are represented by black dotted lines. Transparent spheres highlight stacking interactions between protein residues and the corresponding bases. (**a**,**d**) show the initial situation; and (**b**,**e**) after 20 ns of simulation of 5'-A and 5'-C terminated hAgo2 bound guide strands, respectively; (**c**,**f**) superposition of (**a**,**b**); and (**d**,**e**) with neighboring protein residues at t = 0 ns are shown in green and at t = 20 ns in magenta.
